# Retraction Note: Successful implementation of an enhanced recovery after surgery (ERAS) protocol reduces nausea and vomiting after infratentorial craniotomy for tumour resection: a randomized controlled trial

**DOI:** 10.1186/s12883-020-02027-1

**Published:** 2020-12-17

**Authors:** Dan Lu, Yuan Wang, Tianzhi Zhao, Bolin Liu, Lin Ye, Lanfu Zhao, Binfang Zhao, Mingjuan Li, Lin Ma, Zhengmin Li, Jiangtao Niu, Wenhai Lv, Yufu Zhang, Tao Zheng, Yafei Xue, Lei Chen, Long Chen, Xude Sun, Guodong Gao, Bo Chen, Shiming He

**Affiliations:** 1 Department of Neurosurgery, Xi’an International Medical Center, Xi’an, China; 2 Department of Neurosurgery, Tangdu Hospital, The Fourth Military Medical University, Xi’an, China; 3 Department of Neurosurgery, Shaanxi Provincial People’s Hospital, Xi’an, China; 4 Department of Anesthesiology, Tangdu Hospital, The Fourth Military Medical University, Xi’an, China; 5 Department of Nutrition, Tangdu Hospital, The Fourth Military Medical University, Xi’an, China

**Retraction Note: BMC Neurology 20, 150 (2020)**

**https://doi.org/10.1186/s12883-020-01699-z**

The Editor has retracted this article [1] because upon investigation the authors have been unable to provide documents confirming that ethics approval was obtained prior to study commencement. Further, numerous discrepancies have been found between the trial registration and the published article regarding recruitment dates, number of participants being recruited and age of participants. Finally, the authors have informed the journal that they included control data collected as part of a different trial. As such, the editor is no longer confident in the reliability of the data underlying the conclusions.

Author Dan Lu has stated on behalf of all co-authors that they agree to this retraction.
